# The accuracy of FAST in relation to grade of solid organ injuries: A retrospective analysis of 226 trauma patients with liver or splenic lesion

**DOI:** 10.1186/1471-2342-9-3

**Published:** 2009-03-26

**Authors:** Beat Schnüriger, Joachim Kilz, Daniel Inderbitzin, Miranda Schafer, Ralph Kickuth, Martin Luginbühl, Daniel Candinas, Aristomenis K Exadaktylos, Heinz Zimmermann

**Affiliations:** 1 Department of Visceral and Transplantation Surgery, Bern University Hospital, Bern, Switzerland; 2 Department of Trauma and Emergency Medicine, Bern University Hospital, Bern, Switzerland; 3 Department of Diagnostic, Interventional and Pediatric Radiology, Bern University Hospital, Bern, Switzerland; 4 Department of Anaesthesia, Bern University Hospital, Bern, Switzerland

## Abstract

**Background:**

This study investigated the role of a negative FAST in the diagnostic and therapeutic algorithm of multiply injured patients with liver or splenic lesions.

**Methods:**

A retrospective analysis of 226 multiply injured patients with liver or splenic lesions treated at Bern University Hospital, Switzerland.

**Results:**

FAST failed to detect free fluid or organ lesions in 45 of 226 patients with spleen or liver injuries (sensitivity 80.1%). Overall specificity was 99.5%. The positive and negative predictive values were 99.4% and 83.3%. The overall likelihood ratios for a positive and negative FAST were 160.2 and 0.2. Grade III-V organ lesions were detected more frequently than grade I and II lesions. Without the additional diagnostic accuracy of a CT scan, the mean ISS of the FAST-false-negative patients would be significantly underestimated and 7 previously unsuspected intra-abdominal injuries would have been missed.

**Conclusion:**

FAST is an expedient tool for the primary assessment of polytraumatized patients to rule out high grade intra-abdominal injuries. However, the low overall diagnostic sensitivity of FAST may lead to underestimated injury patterns and delayed complications may occur. Hence, in hemodynamically stable patients with abdominal trauma, an early CT scan should be considered and one must be aware of the potential shortcomings of a "negative FAST".

## Background

A fast diagnostic workup with high accuracy is an important prerequisite for the successful management of patients with multiple injuries [1]. The acronym "FAST" (Focused Assessment with Sonography for Trauma) first appeared in 1995 and the detailed technique was defined at the International Consensus Conference, 1997 as real-time sonographic scanning for free fluid in 4 distinct regions of the torso: the pericardial, perihepatic, perisplenic, and pelvic regions (2, 3). FAST has reached worldwide importance through its incorporation into the algorithms of Advanced Trauma Live Support^® ^(ATLS^®^) [2]. But, the role of FAST must be continuously reassessed because, despite its high specificity, ultrasonography (US) has a low sensitivity ranging from 40–80% for the detection of free fluid and particularly of organ lesions [3-6]. Furthermore, non-operative management of hemodynamically stable patients with liver or splenic lesions has become the standard of care [7,8]. This significant change in the therapeutic algorithm and the poor diagnostic power of FAST has led us to reconsider the clinical relevance of our diagnostic effort [9].

The computed tomography (CT) scan of the abdomen is currently considered the gold standard for detecting intra- and retroperitoneal lesions in trauma patients [10-13]. According to ATLS^®^, an abdominal CT scan is indicated in hemodynamic normal trauma patients with impaired sensorium (brain injury, alcohol, drugs), and equivocal abdominal findings [2].

However, trauma centers are equipped with dedicated CT scanners to allow fast access to emergency patients, especially those with multiple injuries [14]. Scanning times of 8 minutes are realistic and first interpretation can be performed 16 minutes after arrival of the patient in the examination room and 35 minutes after admission in the ED, respectively [15]. In our trauma facility, an intravenous contrast-enhanced multiple trauma CT scan (i.e. Head, thorax, abdomen and pelvis) requires an average of 25 (range 13–49) minutes to complete [16].

The aim of the present study was to investigate the role of FAST in the diagnostic algorithm in multiply injured patients in a modern ED with immediate access to a CT scanner. Therefore, we analysed the results of FAST in 226 multiply injured patients with liver or splenic lesions in relationship to the grade of organ injury. Additionally, we characterized the FAST-false-negative patients and determined the clinical consequences of a false-negative FAST.

## Methods

The study was conducted at the Bern University Hospital, Switzerland between January 2001 and July 2006. An average of 286 (range, 204–344) multiple injured patients are treated in our level I trauma centre during this time each year [17].

Immediately after primary assessment of the patient by the attending surgeon, a senior resident in radiology performs bedside the FAST to detect free fluid (Hitachi^® ^EUB-6500). In hemodynamically stable patients with the history of a blunt or penetrating abdominal trauma, impaired sensorium, and unclear abdominal clinical findings, a contrast enhanced helical abdominal CT scan (Siemens^® ^Somatom Sensation 16) is conducted.

Hemodynamically stable patients with (1) a FAST examination on admission, and (2) a spleen or liver lesion documented by (3) an abdominal intravenous contrast enhanced helical CT scan were included in this study. A total of 226 patients fulfilled these three criteria.

In the first step, the original radiological reports of the FAST and CT scans, and inpatient records were systematically reviewed. Data concerning the mechanism of injury and accompanying extra- and intra abdominal injuries were collected.

The injury severity score (ISS), according to Baker et al., was calculated and the Abbreviated-Injury-Scale (AIS) grading for chest and neurological trauma was used [18,19]. The scale devised by the Organ Injury Scaling Committee of the American Association for the Surgery of Trauma was used to grade injuries to the spleen, liver, and kidney [20,21].

Free intra-abdominal fluid or liver and/or spleen lesions detected by FAST were defined as FAST-positive. Free intra-abdominal fluid or organ lesions detected by CT scan, but not by FAST, were defined as FAST-false-negative.

The sensitivity and specificity of FAST was then calculated. We further determined the diagnostic accuracy of FAST in relationship to the severity of the organ lesions as depicted by contrast enhanced helical CT scan according to Mirvis et al [22,23].

Statistical calculations were performed using SigmaStat 1.0 (Jandel Scientific Corp., Germany). Means (ISS, age) were compared using the Mann-Whitney test, and proportions using the Chi-square and Fischer exact test. A value of *p *< 0.05 was considered significant.

## Results

### Patient characteristics

A total of 164 male and 62 female multiply injured patients with liver or spleen injuries were included in this study. The mean age of the study cohort was 38 years (SD ± 15.0 years). The mean ISS was 17.8 (SD ± 6.9). A splenic lesion was found in 98 patients (44%), a hepatic lesion in 87 patients (38%), and the combination of splenic and hepatic lesions was found in 41 patients (18%).

### Overall accuracy of FAST

In 45 of 226 patients with CT-confirmed spleen or liver injuries, the initial FAST failed to detect free fluid or organ lesions (sensitivity 80.1%). FAST showed free fluid without confirmation in the CT examination in one patient with a grade II liver contusion and a subcapsular hematoma (specificity 99.5%). The overall positive and negative predictive values were 99.4% and 83.3%, respectively. The overall likelihood ratios for a positive and negative FAST were 160.2 and 0.2, respectively. Of 41 patients with a combination of hepatic and splenic lesions, FAST was false negative in 7 cases (sensitivity 82.9%). The diagnostic accuracy of FAST was identical for splenic and hepatic injuries (Fisher exact test: *p *= n.s.).

### FAST in correlation with the CT findings

Table 1 summarizes the accuracy of FAST in relationship to the CT based grading of spleen or liver injuries. Grade III-V lesions were more reliably identified by FAST then grade I and II lesions (Fisher exact test: spleen: *p *= 0.0077, liver: *p *= 0.0081). In 21 of 53 patients with grade I hepatic or splenic lesions, FAST could not detect free fluid or any organ lesion (sensitivity 60.4%). In grade II lesions, the sensitivity was 78.6%, and in grade III lesions it was 88.6%. In 32 patients with grade IV and V spleen or liver injuries, FAST could always detect either free fluid or directly demonstrate the organ lesion (sensitivity 100%).

**Table 1:** The sensitivity of FAST in relation to the severity of a spleen or liver injury

Spleen lesion Grade	n =	FAST false negative (n =)	%-FAST false negative	Sensitivity of FAST	Liver lesion Grade	n =	FAST false negative (n =)	%-FAST false negative	Sensitivity of FAST
	
I	26	9	34.6%	65.4%	I	27	12	44.4%	55.6%
	
II	26	6	23.1%	76.9%	II	30	6	20.0%	80.0%
	
III	24	3	12.5%	87.5%	III	20	2	10.0%	90.0%
	
IV	18	0	0.0%	100.0%	IV	9	0	0.0%	100.0%
	
V	4	0	0.0%	100.0%	V	1	0	0.0%	100.0%
	
**I-V**	**98**	**18**	**18.4%**	**81.6%**	**I-V**	**87**	**20**	**23.0%**	**77.0%**

FAST: Focused Assessment with Sonography for Trauma

Of the 7 FAST-false-negative patients with a combination of splenic and hepatic lesions, two had a grade III liver injury in combination with a grade I spleen injury. The five other patients had combinations of grade I and II hepatic and splenic lesions.

### 4. Characteristics of the FAST-false-negative patients

The mean age of the FAST-false-negative patients was 42 years (SD ± 19.1 years) and 37 years (SD ± 19.0 years) in the FAST-positive group (Mann-Whitney test: *p *= n.s.). There was no difference in the gender ratio between these two groups (Chi-square test: *p *= n.s.).

Table 2 shows the mechanisms of injury in the FAST-false-negative patients. In summary, 32 of our 45 FAST-false-negative patients had a traffic accident with either a high-velocity or a low velocity mechanism with crush and prolonged rescue times, or bicycle accidents. A total of 9 patients suffered falls ≥ 2.5 m.

**Table 2:** Mechanism of injury in FAST-false-negative patients

Mechanism of injury in the "FAST-false-negative" patients	n	%
Car accident (High velocity; low velocity with compression or crushing injury; car ejection injury)	15	33%

Motorcycle accident	10	22%

Fall of ≥ 2.5 m	9	20%

Bicycle accident	7	17%

Skiing-/snowboarding accident	2	4%

Stab wound	1	2%

Unknown	1	2%

Total	45	100%

FAST: Focused Assessment with Sonography for Trauma

The ISS of the FAST-false-negative and FAST-positive patients in relation to the injured organ (spleen or liver) is shown in Table 3 (Mann-Whitney test: *p *= n.s.). The over-all mean ISS of the FAST-false-negative patients was 17.6 (SD ± 10.0). Without the additional diagnostic benefit of a CT scan, the mean ISS of these patients would have been 13.0 (SD ± 10.1), which would significantly underestimate the actual severity of their injuries (Mann-Whitney test: *p *= 0.0095).

**Table 3:** ISS of FAST-false-negative and FAST-positive patients in relation to the injured organ

Injured organ(s)	Median ISS of the FAST-false-negative patients (n = 45)	Median ISS of the FAST-positive patients (n = 181)	p-value*
Spleen (n = 98)	14.0 (SD ± 6.7) (n = 18)	16.0 (SD ± 9.0) (n = 80)	0.425

Liver (n = 87)	14.0 (SD ± 9.4) (n = 20)	17.1 (SD ± 9.0) (n = 67)	0.196

Spleen and Liver (n = 41)	30.3 (SD ± 9.6) (n = 7)	24.3 (SD ± 7.8) (n = 34)	0.213

*Mann-Whitney Test

FAST: Focused Assessment with Sonography for Trauma

ISS: Injury severity score

Additional and previously unsuspected intra-abdominal injuries detected by CT scans in the FAST-false-negative patients included: 2 renal contusions (grade II), 1 grade III renal laceration, 2 hemorrhages of the suprarenal gland, 1 colonic perforation, and 1 retroperitoneal hematoma. Of note, surgical intervention was only needed in the patient with colonic perforation. The other incidental findings could be treated conservatively.

Further extra-abdominal severe injuries in the 45 FAST-false-negative patients included: 25 (56%) severe thoracic injuries (AIS grade 3 and 4), 8 (18%) severe acute brain injuries (AIS grade 3, 4 and 5), and 5 (11%) unstable pelvic fractures.

## Discussion

This clinical study implies that the FAST examination at the primary assessment fails to detect free fluid or organ lesions in 1 of every 5 patients with confirmed spleen or liver injury. On closer examination, it also shows that higher grade lesions were significantly more likely to be identified by FAST than lower grade lesions. This disparity in sensitivity is nicely shown in Table 1. The sensitivity of FAST in grade IV and V splenic or liver lesions was 100%. Hence, FAST is an expedient but rough tool for the primary assessment of hemodynamically unstable, polytraumatized patients with grade IV and V lesions. In this situation, the attendant surgeon should consider an immediate laparotomy without further CT scan. However, the majority of patients, even those with high grade lesions, respond to fluid replacement therapy and in settings with immediate access to a CT scanner, the role of FAST should be reconsidered.

With an exceptional likelihood ratio of a positive result (160.2) FAST is an excellent test when positive. However, the inadequate likelihood ratio of a negative test (0.2) emphasizes the risk to miss intra-abdominal injuries. According to the literature, 11–34% of patients with even high grade spleen and liver injury show no evidence of hemoperitoneum and therefore may appear FAST negative [4,24]. Morbid obesity or severe subcutaneous emphysema can increase the rate of FAST-false-negative results. An important disadvantage of sonography is the poor assessment of the retroperitoneal space and the unreliable detection of free intraperitoneal air [1]. In our FAST-false-negative group, this resulted in initially undiagnosed renal and suprarenal injuries and in one patient to an emergency laparotomy due to an unrecognised colon perforation. Incidental CT findings in polytraumatized patients vary in their surgical importance, but must be expected in up to 17% of cases and are typically located in the abdomen [25,26]. Pathological findings in the pelvis or chest x-ray seem to be superior predictors for a positive abdominal CT scan in blunt trauma patients [27]. The representative injury pattern in the FAST-false-negative patients in our series included severe thoracic injury in 56%, severe acute traumatic brain injury in 18%, and unstable pelvic fractures in 11% of cases. Hence, the majority of our FAST-false-negative patients presented with an indication for an abdominal CT scan.

The mechanism of injury alone doesn't seem to be a predictive factor for a positive abdominal CT scan too [27,28]. However, based on the data obtained, we advocate that every patient involved in high velocity traffic or crush accident should be considered a candidate for an additional CT scan.

Of note, 2 patients with grade II splenic lesions and negative initial FAST required a haemostatic splenorrhaphy and a splenectomy due to delayed haemorrhage (on days 1 and 11, respectively, after trauma). From the clinical point of view, it is crucial to thoroughly detect all abdominal injuries early. Even low grade lesions can be the source of relevant (typically delayed) bleeding [29,30].

Currently, the initial CT diagnostic workup is considered the gold-standard for the systematic evaluation of the polytraumatized patient [10-13]. The overall sensitivity of the CT scan is >95% for intra- and retroperitoneal solid organ lesions after trauma [12,31]. Typically, in high-grade hepatic and splenic lesions, active haemorrhage and the development of traumatic pseudoaneurysms is observed [29,32-34]. Only the initial CT scan can precisely determine the extent and pattern of parenchymal and vascular injury, and allows for an individual decision for early surgical or endovascular repair when appropriate [33]. A repetitive CT assessment of high grade parenchymal injuries permits successful non-operative management of blunt and even selected penetrating injuries in the majority of cases [35,36].

## Conclusion

FAST is an excellent test when positive. However, the inadequate likelihood ratio of a negative test (0.2) emphasizes the risk to miss intra-abdominal injuries, which has led to a significant underestimation of the injury pattern. High grade lesions can be detected reliably by FAST, but only a CT scan can determine the extent and pattern of parenchymal and vascular injury, and therefore allow for individually tailored and often non-operative therapeutic management. Hence, in hemodynamically stable patients with abdominal trauma, an early CT scan should be considered, and one must be aware of the potential shortcomings of a so-called "negative FAST".

## Limitations

The major limitations of this study are its retrospective design and potential for selection bias. The diagnostic capability of ultrasonography depends on the skill and experience of the examiners. Furthermore, with only a few patients with grade IV and V splenic and liver injuries included in this series, our conclusions are cautious.

## Competing interests

The authors declare that they have no competing interests.

## Authors' contributions

BS coordinated the statistical analysis, interpreted the results and drafted the manuscript. JK collected the radiological data and performed the statistical analysis. DI participated in the interpretation of data and draft of the manuscript. MS collected the clinical data and performed the statistical analysis. RK and ML revised the manuscript critically for radiological and emergency medicine contents. DC gave final approval of the version to be published. AK conceived, designed and coordinated the study. HZ gave final approval of the version to be published.

## Pre-publication history

The pre-publication history for this paper can be accessed here:

http://www.biomedcentral.com/1471-2342/9/3/prepub
